# Supplementary material for: Impact of Delta SARS-CoV-2 Infection on Glucose Metabolism: Insights on Host Metabolism and Virus Crosstalk in a Feline Model
Source: Viruses. 2024 Feb 15;16(2):295. doi: 10.3390/v16020295 (PMC10893195; doi:10.3390/v16020295)
Supplement: Supplementary file 1 [file viruses-16-00295-s001.zip › viruses-2843141-supplementary.pdf]

Table S1: List of primers and probe sequences used for ddPCR.

Oligo name	Sequence
2019-nCoV_N1-Forward	GACCCCAAAATCAGCGAAAT
2019-nCoV_N1-Reverse	TCTGGTTACTGCCAGTTGAATCTG
2019-nCoV_N1-Probe	/56-FAM/ACCCCGCAT/ZEN/TACGTTTGGTGGACC/3IABkFQ/
2019-nCoV_N2-Forward	TTACAAACATTGGCCGCAAA
2019-nCoV_N2-Reverse	GCGCGACATTCCGAAGAA
2019-nCoV_N2-Probe	/56-FAM/ACAATTTGC/ZEN/CCCCAGCGCTTCAG/3IABkFQ/
RNAseP_Forward	AGATTTGGACCTGCGAGCG
RNAseP_Reverse	GAGCGGCTGTCTCCACAAGT
RNAseP_Probe	/56-FAM/TTCTGACCT/ZEN/GAAGGCTCTGCGCG/3IABkFQ/
ACE2 Forward	ACGGAGGCGTAAGGATTT
ACE2 Reverse	GTGTGGTAGTGGTTGGTATTG
ACE2 Probe	CGGATCAGAAATCGAAGG-AAGAA

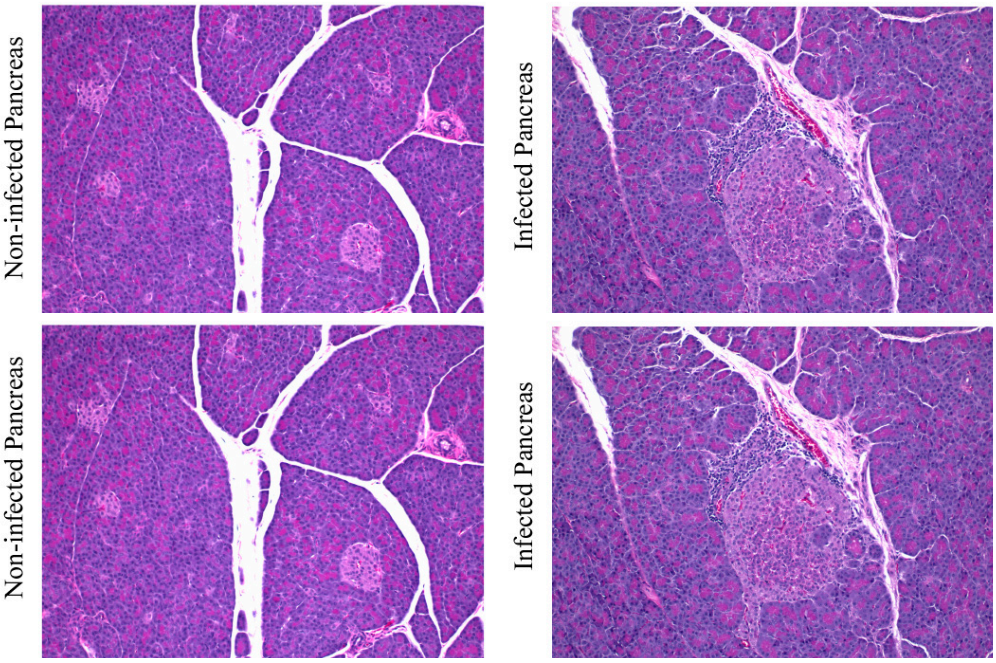

Figure S1. *Lymphoplasmacytic inflammation affecting one large islet at 12 dpi.* H&E images of pancreas tissue with islets from a healthy non-infected control cat (A) vs. one SARS-CoV-2-infected cat (B) that exhibited small lymphocytes and plasma cell infiltrates disrupting the normal architecture of a large islet at 12 dpi (B). No other histopathologic findings were observed in the pancreas of infected cats.
